# Considering vulnerable communities in climate mitigation and adaption plans, India 

**DOI:** 10.2471/BLT.22.288846

**Published:** 2022-11-09

**Authors:** Adithya Pradyumna, Sheetal Patil, Madhuri Ramesh

**Affiliations:** a School of Development, Azim Premji University, Survey No 66, Burugunte Village, Bikkanahalli Main Road, Sarjapura, Bengaluru 562125, India.; b School of Environment and Sustainability, Indian Institute for Human Settlements, Bengaluru, India.

The nature and magnitude of the impacts of climate change on human health have become clearer in recent times. The effects mediated through the disruptions to biodiversity and agroecosystems are of particular concern in several contexts. Climatic hazards such as floods and droughts can worsen food insecurity and malnutrition in vulnerable human populations.^1^ In India, governmental policy and programmatic interventions for biodiversity, agriculture and health increasingly recognize and reflect the importance of climate change mitigation and adaptation.^2^^,^^3^

While this policy attention is necessary and timely, we examine some of the common approaches used to address climate change in India using a climate justice lens. In other words, we describe how the cost of popular mitigation and adaptation measures are sometimes disproportionately borne by marginalized communities, further jeopardizing their already vulnerable situation. This pattern of injustice and disproportionate costs was also observed during the coronavirus disease 2019 pandemic, where vulnerable populations had more difficulties in accessing food, livelihood and health care due to the abrupt movement restrictions instituted across India.^4^ Using examples of projects on renewable energy, afforestation and coastal embankments, we briefly describe how the unintended negative health impacts of these interventions are mediated through agriculture and biodiversity, alongside other pathways. We argue that active recognition of injustices in climate action is a crucial step towards designing equitable and fair responses to large-scale environmental crises. Doing so is important to countries such as India, where a large proportion of the population is already vulnerable to climate change and endures poor health outcomes.

## Renewable energy

Solar and other renewable energy is a critical part of the solution to climate change. Solar energy has received substantial attention in India’s National Action Plan on Climate Change through the National Solar Mission.^2^ The National Institute for Solar Energy estimates that the solar energy potential in India is about 750 gigawatts, based on 3% of land use classified as wastelands that could be made available for the development of these projects. However, due to the remote location of these wastelands and high costs involved in connecting them with the power grid, proposals to install large-scale solar power plants have been shifted to other lands. This action has led to diversion of land from agriculture, forests and other uncultivated landscapes, giving rise to new socioecological challenges.^5^

The socioecological footprint of the large-scale solar energy generation projects is reported to be as massive and destructive as large hydroelectric power projects.^5^ Early impacts include displacement of critical wildlife and human communities as well as degradation of soil and water resources. For instance, pastoralist communities in the Kutch region were displaced from their traditional grazing lands, and farmers in Pavagada subdistrict of Karnataka State were economically displaced from their farmlands because their resources, such as seasonal cultivation opportunities that would have contributed to their subsistence food demands, were compromised. After the solar park was installed, loss of pollinator diversity was observed in adjacent farmlands, reportedly resulting in a decline in crop yield.^6^ This effect might have far-reaching consequences, since insect-pollinated crops form an important part of the country’s economy.

Use of large volumes of water (mostly groundwater) for washing the solar panels is another cause of concern for farmers in the surrounding semi-arid landscape. In some situations, the electricity generated from these farmland-based projects has not led to electrification of local homes.^6^ A combination of these factors have implications for nutrition, mental health and access to health care. These implications are worrisome since rural communities are already distressed and a high prevalence of conditions such as undernutrition exists in many parts of rural India.^7^

## Afforestation

Afforestation is a long-standing activity in India that has more recently been explicitly linked to climate change mitigation, towards creating a sink for over 2.5 gigatons of carbon dioxide.^8^ However, afforestation activities have been conducted in a way that has adversely affected disadvantaged communities in several situations. Lands with old growth forests and rich biodiversity continue to be diverted for industrial and infrastructure projects,^8^ affecting communities that are directly dependent on them for livelihood, nutrition, medicine and culture.^9^ Deforestation also adds to challenges such as vector-borne diseases through the increase in vector-breeding sites and exposure to disease vectors.^10^ To compensate for the loss of forests, afforestation projects often plant monocultures of fast-growing trees,^8^ in some situations on lands that belong to indigenous communities.^11^ Doing so can further compromise the nutrition and well-being of indigenous communities.

Afforestation activities are also taken up as part of watershed development projects to improve soil and moisture conservation on degraded and so-called wastelands in over 2 million square kilometres in India, by cordoning lands to allow trees to grow.^12^ On one hand, such measures certainly help to improve long-term water security, which in turn can contribute to sanitation and nutrition-related outcomes. On other hand, these measures can negatively affect landless households that are dependent on uncultivated common lands to feed their livestock,^7^ which are critical for the nutrition and well-being of these households. Policy guidelines now explicitly recognize the need to support the livelihood of landless households.^12^ However, the implementation of these equity aspects has been patchy.^7^

## Coastal embankments

In coastal areas, an important impact of climate change is the erosion of land and ingress of saline water triggered by a rise in the sea level. This development could be accompanied by increased flooding. For instance, in the Sundarbans in eastern India, repeated bouts of rains and floods have degraded agricultural land and destroyed kitchen gardens and thereby eroded the food security of these rural communities. Moreover, the ingress of saline water has affected inland fisheries, another important source of food. The prevalence of waterborne diseases has also increased.^13^ In response, governmental interventions have typically focused on extending embankments – that is, sea walls – along the Sundarbans coast for almost 3000 km, using concrete to make them robust.^14^ However, since concrete is an impermeable material, it traps rainwater on the landward side during storms and cyclones, and thereby increases flooding. The higher risk of flooding could, in turn, exacerbate the nutritional and other health problems faced by these communities.

In addition, embankments cut off access to the sea, further burdening fishing communities who already struggle with the challenges posed by climate change. For example, fishing communities in the southern State of Kerala have suffered economic losses due to climate change (for instance, decline in sardine catch, loss of fishing days due to cyclones) as well as loss of homes and property due to accelerated erosion of the shoreline.^15^ In this case, the construction of embankments in adjoining areas has disrupted sediment flow along the coastline and increased erosion at the fishing villages. Over half of the State’s coastline has embankments; therefore, the impact on fishers is important.^15^ Overall, these structures become maladaptive since they are designed and constructed without sufficient attention to local geographical and social needs. Such interventions could create climate refugees among both agricultural and fishing communities in those geographies.

## Conclusions

Vulnerable and marginalized communities have disproportionately borne both the impacts of climate change and the unintended negative impacts of action addressing climate change. This situation is unacceptable. Action against climate change, just like any developmental action, needs to be attentive to the rights of vulnerable communities, and ensure a fair distribution of both burdens and benefits of interventions. While national-level policies are important, especially for mitigation, we suggest enhancing decentralized approaches to climate action with strong community engagement to facilitate equitable outcomes. All the stages of interventions, from planning to evaluation, should involve multiple stakeholders, especially at local level. One way in which this can be operationalized is through conducting social and health impact assessments for planned interventions, even for those that appear to be purely for the benefit of local residents. If conducted well and given due importance, such interventions could help with early course correction to prevent unintended and inequitable distribution of any potential negative impacts.^6^ Doing so may also appropriately influence the nature of interventions themselves, for instance, through ecosystem-based adaptation. An example of such an intervention would be small- and medium-scale solar energy cooperatives in combination with agrivoltaic regimes, which would foster both food and energy sovereignty of small landholders.^16^ Similarly, a need exists for better regulation of infrastructure development in sensitive geographies, for example on seafronts. If such interventions can be combined with better access to housing, health care, nutrition and livelihoods, the vulnerability of communities such as fisherfolk could be better addressed. In India, the National Action Plan on Climate Change and Human Health alludes to the importance of community participation and intersectoral action to manage the health effects of climate change,^3^ but much remains to be done. The vulnerable should not have to pay again for action against climate change, having already unjustly paid a heavy price thus far for the impacts of climate change and environmental degradation.
